# A novel method for liquid-phase extraction of cell-free DNA for detection of circulating tumor DNA

**DOI:** 10.1038/s41598-021-98815-x

**Published:** 2021-10-04

**Authors:** Filip Janku, Helen J. Huang, David Y. Pereira, Masae Kobayashi, Chung Hei Chiu, S. Greg Call, Kristen T. Woodbury, Felix Chao, Daniel R. Marshak, Ricky Y. T. Chiu

**Affiliations:** 1 Department of Investigational Cancer Therapeutics (Phase1 Clinical Trials Program)–Unit 455, The University of Texas MD Anderson Cancer Center, 1515 Holcombe Blvd, Houston, TX 77030 USA; 2 Phase Scientific International Ltd, Garden Grove, CA USA; 3 Phase Scientific International Ltd, Kwun Tong, Hong Kong

**Keywords:** Tumour biomarkers, Cancer

## Abstract

Low yields of extracted cell-free DNA (cfDNA) from plasma limit continued development of liquid biopsy in cancer, especially in early-stage cancer diagnostics and cancer screening applications. We investigate a novel liquid-phase-based DNA isolation method that utilizes aqueous two-phase systems to purify and concentrate circulating cfDNA. The PHASIFY MAX and PHASIFY ENRICH kits were compared to a commonly employed solid-phase extraction method on their ability to extract cfDNA from a set of 91 frozen plasma samples from cancer patients. Droplet digital PCR (ddPCR) was used as the downstream diagnostic to detect mutant copies. Compared to the QIAamp Circulating Nucleic Acid (QCNA) kit, the PHASIFY MAX method demonstrated 60% increase in DNA yield and 171% increase in mutant copy recovery, and the PHASIFY ENRICH kit demonstrated a 35% decrease in DNA yield with a 153% increase in mutant copy recovery. A follow-up study with PHASIFY ENRICH resulted in the positive conversion of 9 out of 47 plasma samples previously determined negative with QCNA extraction (all with known positive tissue genotyping). Our results indicate that this novel extraction technique offers higher cfDNA recovery resulting in better sensitivity for detection of cfDNA mutations compared to a commonly used solid-phase extraction method.

## Introduction

Isolation of cell-free DNA from blood or other body fluids from patients with cancer has revolutionized molecular cancer diagnostics and cancer care^1^. Short fragments of double-stranded cell-free DNA (cfDNA) are released to the circulation from cells that have undergone apoptosis or necrosis. These fragments can be isolated and utilized for detection and molecular profiling of the circulating tumor DNA (ctDNA) fraction. Previously, our group at the University of Texas MD Anderson Cancer Center has developed several high-sensitivity molecular diagnostic techniques for cfDNA mutation detection^2,3^. These approaches utilized solid-phase nucleic acid extraction methods and downstream droplet digital PCR (ddPCR) or next-generation sequencing (NGS) diagnostic platforms. One key limiting factor for broader utilization of cfDNA based liquid biopsies in cancer is the relatively small amount of cfDNA and ctDNA that can be obtained using existing isolation techniques^4^. In advanced cancers, typical isolation yields are between 15–20 ng of cfDNA per 1 mL of plasma using commonly used cfDNA extraction kits^5,6^. In patients with localized cancers, isolation yields are lower, often not exceeding 5 ng per mL of plasma, which limits its utility for applications such as detection of minimal residual disease, monitoring the efficacy of adjuvant therapy, or early cancer screening^7,8^. Collectively, this represents an unmet need for novel sample preparation methods that offer improved cfDNA and ctDNA recovery from plasma.

The industry standard for nucleic acid sample preparation (including cfDNA) utilizes solid-phase extraction methods such as silica matrix or paramagnetic bead technology for purification. These techniques have been proven to achieve high recovery and purity of total nucleic acids; however, they have significant limitations in capturing minute levels of DNA and particularly low molecular weight DNA, which is a typical characteristic of cfDNA^9^. To address the need for better cfDNA isolation and purification techniques, we investigated a liquid-phase extraction method for isolation and purification of cfDNA called PHASIFY™ (Phase Scientific International Limited, Kwun Tong, Hong Kong). The method is based on aqueous two-phase systems (ATPSs) that consist of polymer, salt and/or surfactant components that intrinsically separate into two distinct phases. Previously, ATPSs have been used to partition DNA, typically plasmid or genomic DNA as a bioseparation technique^10,11^. The PHASIFY method adopts the bioseparation capability of ATPSs for cancer diagnostic utility, resulting in a novel sample preparation platform. The PHASIFY method leverages a series of ATPS extractions that are optimized to drive cfDNA from human plasma into one phase and other components, such as protein contaminants, into the other, achieving both target isolation and sample purification. The volume ratio of the two phases is also manipulated to enable concomitant concentration of the desired analytes (i.e. cfDNA) in the desired phase.

In this study, we report the analytical performance and performance with clinical specimens of the PHASIFY extraction method. First, we compared analytical cfDNA yield from contrived samples between PHASIFY and a commonly used solid-phase extraction method. Next, we extracted cfDNA from cancer patient plasma samples using the PHASIFY method and compared cfDNA yield, mutant copy yield, and overall mutation detection to that of solid-phase extraction.

## Results

### The PHASIFY liquid-phase extraction mechanism

The PHASIFY method utilizes a series of ATPSs with unique, proprietary formulations to isolate, purify, and concentrate cfDNA from a plasma sample. Other groups have utilized ATPSs comprised of surfactants, polymers, salts, and ionic liquids to isolate various biomarkers (including proteins, lipids, nucleic acids, small molecules, and macromolecules)^11–15^. The PHASIFY method takes a similar approach in formulating ATPSs with the specific goal of isolating cfDNA. In the PHASIFY MAX kit, two separate ATPSs are used: the first ATPS serves to purify cfDNA from other dominating plasma components and the second ATPS serves to further purify and concentrate the cfDNA into a small volume.

**Figure 1:**
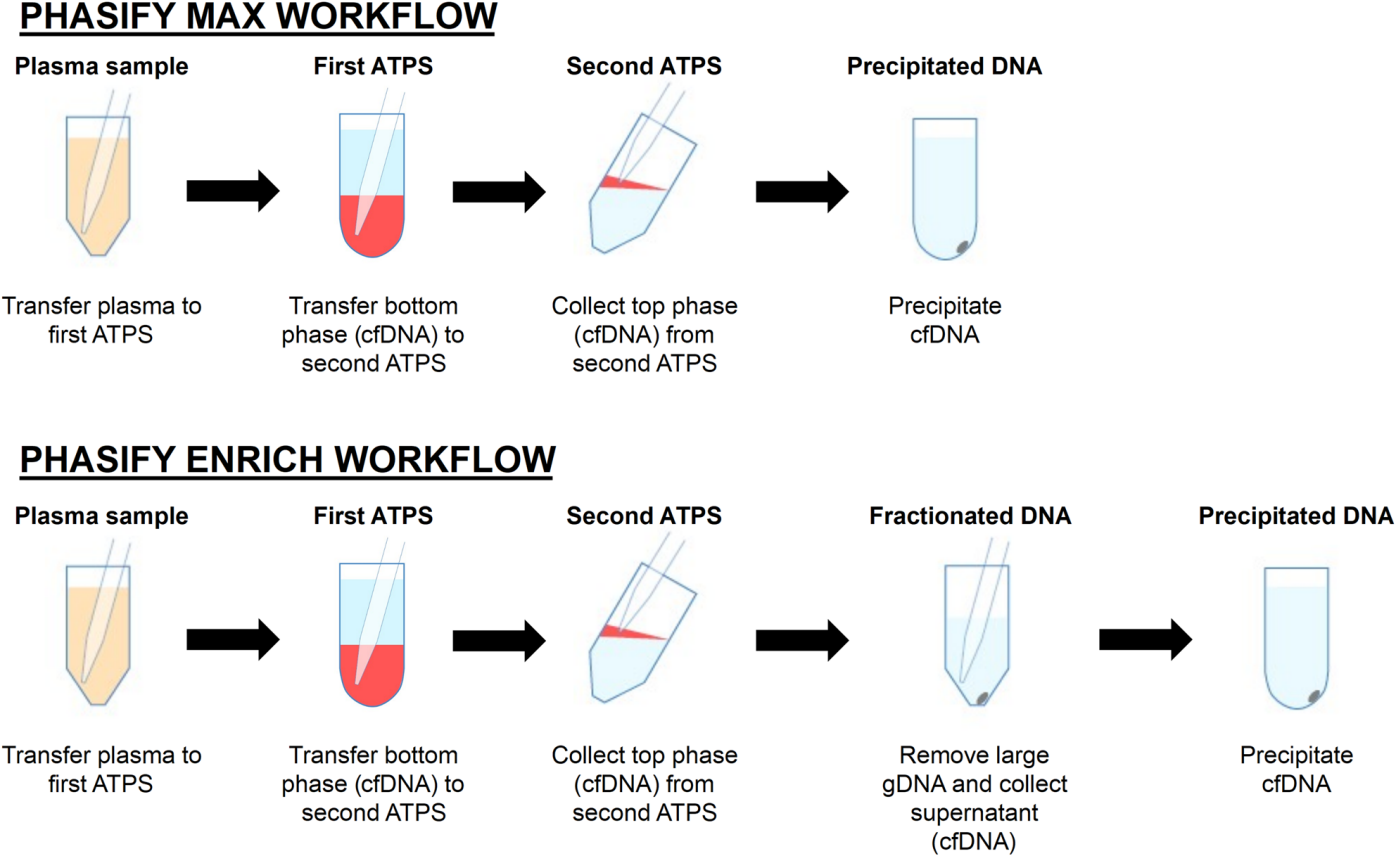
Schematic of DNA isolation process with PHASIFY MAX and PHASIFY ENRICH. The PHASIFY method uses serial two-phase liquid extraction systems to isolate and purify cfDNA from a starting plasma sample. In the first ATPS, DNA partitions to the bottom phase (red), which is then extracted and transferred to a second ATPS. After phase separation, the DNA partitions to the top phase (red), which is then extracted. In the PHASIFY MAX workflow, all extracted DNA undergoes DNA precipitation. In the PHASIFY ENRICH workflow, the extracted DNA is first mixed with a fractionation solution to remove contaminating DNA and enrich the sample with potential tumor cfDNA. The enriched sample then undergoes DNA precipitation.

The first ATPS consists of components that, when mixed with 1 mL of plasma, force the solution to become turbid and undergo phase separation to form a top phase and a bottom phase (Fig. 1). Centrifugation is used to facilitate the phase separation process. After phase separation, cfDNA partitions to the bottom phase and proteins and lipids partition to the top phase due to a carefully optimized combination of electrostatic, hydrophilic/hydrophobic and excluded-volume interactions with the components of the ATPS. The cfDNA-containing bottom phase of the first ATPS is then transferred to a second ATPS with a separate and distinct formulation. In this new system, the cfDNA partitions to the top phase due to optimized attractive van der Walls interactions with the components of the second ATPS. The composition of the second ATPS is formulated so that the top phase-to-bottom phase volume ratio is small (i.e., the top phase volume is smaller in relation to the bottom phase volume). The reduced volume ratio concentrates the purified cfDNA into a small volume. The collected sample then undergoes conventional isopropanol-based precipitation, resulting in a DNA pellet that can be resuspended in a commercial buffer for downstream detection.

The PHASFY ENRICH method, which follows the same workflow as PHASIFY MAX, includes an additional size-selection solution that is used after the second ATPS. The size-selection solution is comprised of similar ATPS components that interact with nucleic acids such that they preferentially precipitate large molecular weight DNA without substantially precipitating small molecular weight cfDNA. The extracted top phase from the second ATPS is mixed with the size-selection solution to initiate the separation of cfDNA based on molecular weight. After an incubation period, the supernatant containing DNA primarily < 500 bp is then transferred and undergoes isopropanol-based precipitation.

### Analytical performance of PHASIFY method

**Figure 2:**
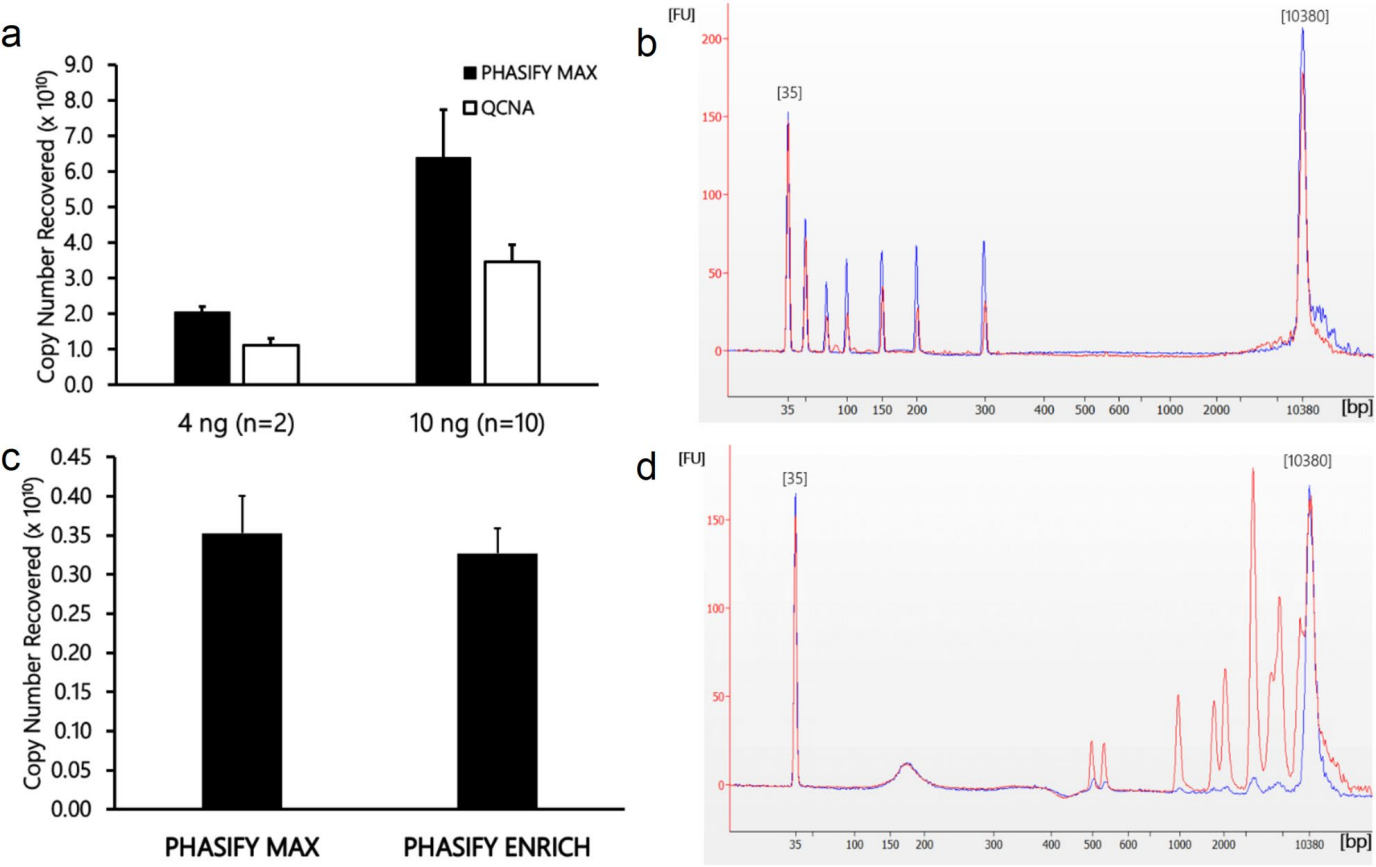
Analytical performance of PHASIFY MAX and ENRICH extraction kits. (**a**) cfDNA recovery of PHASIFY MAX (black bars) was assessed and compared against QCNA (white bars). Four or 10 ng of a synthetic 145 bp dsDNA fragment were spiked into 1 mL healthy human plasma, then processed by both extraction kits. The detection of the recovered cfDNA was performed using Taqman-based probe and primers specific for the 145 bp dsDNA sequence and quantified by ddPCR using a Bio-rad QX200 and QuantaSoft (version 1.7.4; www.bio-rad.com) (**b**) Agilent Bioanalyzer 2100 data demonstrates the ability of both PHASIFY MAX (blue) and QCNA (red) kits to recover all DNA fragment sizes from plasma samples spiked with DNA ladder (50, 75, 100, 150, 200, 300 bp), with PHASIFY-extracted samples obtaining higher peak amplitudes for each fragment size compared to QCNA. (**c**) Contrived samples of 1 ng 145 bp dsDNA spiked into 1 mL of plasma were processed with PHASIFY MAX and PHASIFY ENRICH (n = 3 each). Both kits achieved similar recovery of the 145 bp fragments as detected by ddPCR. (**d**) Plasma spiked with DNA ladder (500 – 10 k bp) were processed with either PHASIFY MAX (red) or PHASIFY ENRICH (blue) extraction kits and the resulting samples were analyzed with an Agilent Bioanalyzer. PHASIFY MAX extracted all fragment sizes contained in the DNA ladder while ENRICH removes larger DNA fragments (> 500 bp).

To assess the analytical cfDNA recovery performance, contrived samples were prepared containing 4 ng or 10 ng of a 145 bp dsDNA in 1 mL healthy human plasma, then subsequently processed with the PHASIFY MAX method. Recovery was quantified by ddPCR and compared with a conventional solid-phase method, QIAamp Circulating Nucleic Acid extraction kit (QCNA; Qiagen, Hilden, Germany). The PHASIFY MAX method recovered 91% more of the 145 bp dsDNA fragment than QCNA from 4 ng/ml samples and 83% more of the 145 bp fragment from 10 mg/ml samples (Fig. 2a). Specifically, for 4 ng samples, 2.1 × 10^10^ ± 1.4 × 10^9^ copies of the cfDNA were recovered by PHASIFY MAX compared to 1.1 × 10^10^ ± 1.8 × 10^9^ copies recovered by QCNA (*P* = *0.029, n* = *2*). For 10 ng samples, PHASIFY MAX yielded 6.4 × 10^10^ ± 1.4 × 10^10^ copies versus 3.5 × 10^10^ ± 4.7 × 10^9^ copies by QCNA (*P* < 0.01). The ability to recover cfDNA of various sizes was assessed using an Agilent Bioanalyzer 2100 (Agilent, Santa Clara, CA USA). Replicates (n = 2) of 1 mL healthy human plasma spiked with GeneRuler Low Range DNA Ladder (Thermo Fisher, Waltham, MA USA) were processed with PHASIFY MAX (Fig. 2b, blue) or QCNA (Fig. 2b, red). The representative DNA fragment profiles demonstrate the ability of both kits to recover all DNA fragments present in the ladder (50, 75, 100, 150, 200, 300 bp). However, PHASIFY MAX-extracted samples obtained higher peak amplitudes for each fragment size compared to QCNA, qualitatively indicating an improvement in small DNA fragment recovery.

To purify the cfDNA further, size selection can be achieved using the PHASIFY ENRICH method, which incorporates an additional liquid separation step to remove high molecular weight contaminating genomic DNA (larger than 500 bp), thereby enriching for cfDNA. Contrived samples containing 1 ng of 145 bp dsDNA in 1 mL healthy human plasma were processed by both PHASIFY MAX and ENRICH extraction kits. Recovery of the 145 bp dsDNA was similar for both kits as detected by ddPCR (Fig. 2c). The ability of the ENRICH method to remove high molecular weight gDNA (> 500 bp) can also be observed in the Bioanalyzer profile (Fig. 2d, blue), whereas PHASIFY MAX retained the larger sized fragments (Fig. 2d, red).

### PHASIFY MAX and ENRICH validation with clinical specimens

Next, we tested the PHASIFY method in 91 clinical plasma samples from 34 unique patients collected at different time points with diverse advanced cancers and *BRAF*, *KRAS* or *NRAS* mutations (Supplementary Table S1). The samples were selected to represent a broad spectrum of tumors with different expected patterns and quantity of ctDNA shedding. Samples were selected from cancers known to shed high amounts of ctDNA (defined by sensitivities of ≥ 70% compared to tumor tissue based on literature or internal data), such as colorectal cancer (36 samples, 40%), non-small cell lung cancer (7, 8%) or cholangiocarcinoma (4, 4%) and cancers expected to shed low amounts of ctDNA (defined by a sensitivities of < 70% compared to tumor tissue based on literature or internal data) such as low-grade serous ovarian cancer (18, 20%), appendiceal cancer (7, 8%), pancreatic cancer (6, 7%) or thyroid cancer (6, 7%)^4,6,16–24^. We isolated cfDNA from 1 mL of plasma using the PHASIFY MAX and PHASIFY ENRICH methods and then compared the two methods to the isolation of cfDNA from 1 mL of plasma using QCNA. Total cfDNA recovery was determined with the Quant-iT PicoGreen dsDNA Assay Kit (Invitrogen, Carlsbad, CA). The ctDNA mutation status was determined using probe-based (*BRAF*, *KRAS*, *NRAS*) droplet digital PCR (ddPCR; Bio-Rad, Hercules, CA).

**Figure 3:**
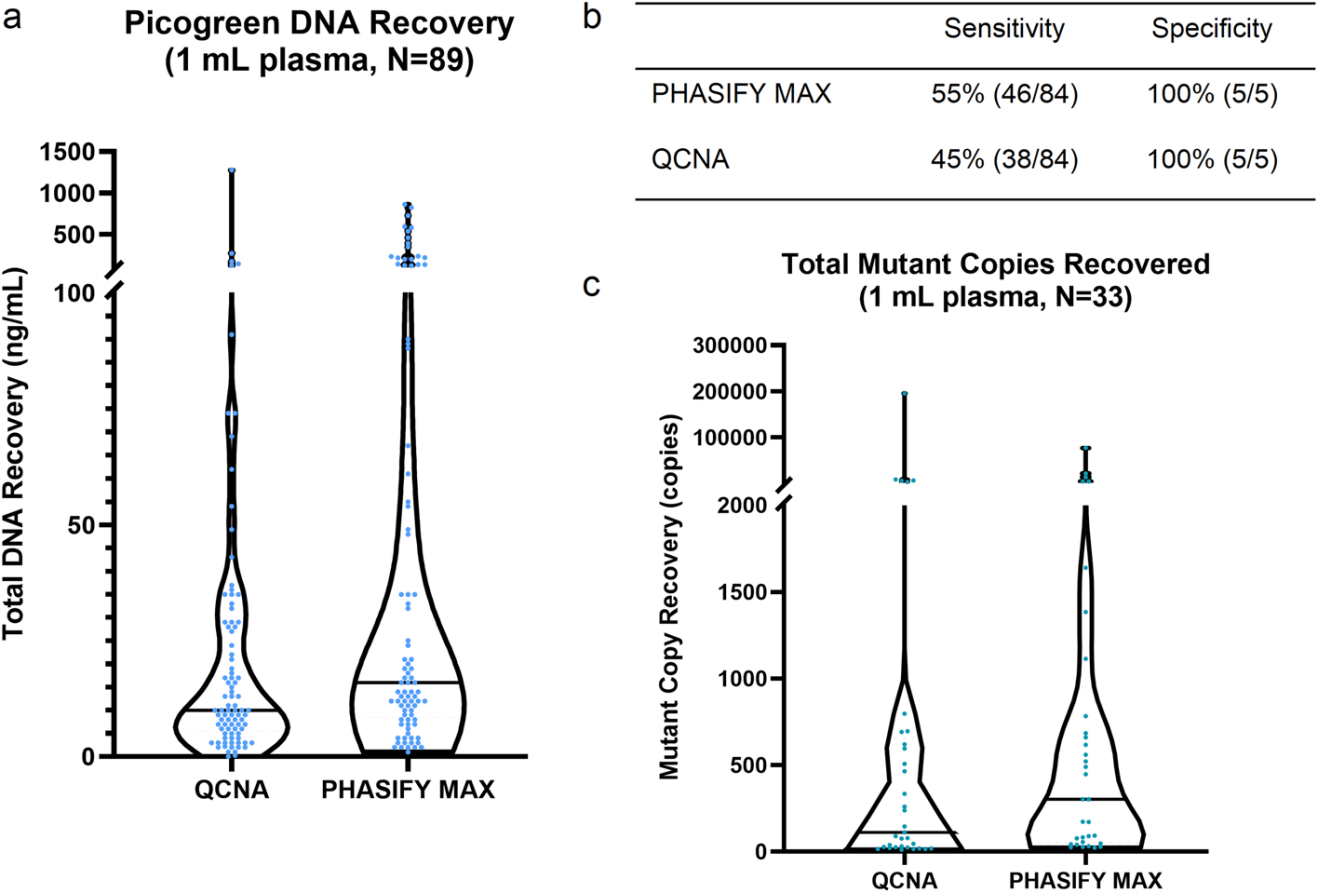
Results of PHASIFY MAX against QCNA. (**a**) A violin plot of total DNA recovery from 1 mL of plasma from QCNA and PHASIFY MAX. Median bars are shown as solid black lines. Median DNA recovery for QCNA and PHASIFY MAX are 10 ng and 16 ng, respectively (60% increase). (**b**) Sensitivity and specificity values of PHASIFY MAX and QCNA against tissue genotyping. (**c**) A violin plot of total mutant copies recovered from QCNA and PHASIFY MAX out of the samples in which both kits detected mutations (n = 33). Median bars are shown as solid black lines. Median mutant copy recovery for QCNA and PHASIFY MAX are 112 copies and 303.6 copies, respectively (171% increase).

The PHASIFY MAX method resulted in isolation of median of 16 ng of DNA (range 1.2–862 ng), which compared favorably to a median of 10 ng of DNA (range 0.3–1,278 ng) obtained with the reference QCNA method (*P* < 0.00001; Fig. 3a). The ddPCR analysis of 89 plasma samples extracted by PHASIFY MAX, which passed quality control testing, demonstrated clinical sensitivity and specificity (against tissue genotyping) of 55% (46/84) and 100% (5/5) respectively and 45% (38/84) and 100% (5/5) respectively with samples extracted by QCNA (Fig. 3b, Supplementary Table S2). Of the samples for which both methods confirmed mutations, on average, PHASIFY MAX achieved a 142% increase in recovery of mutant copies compared to the QCNA procedure. In a comparison of the median in these data, PHASIFY MAX demonstrated a 171% increase compared to QCNA (*P* = 0.0006 Wilcoxon Signed-Rank) (Fig. 3c). In 13 samples, mutations were detected in samples extracted with PHASIFY MAX, but not with QCNA (Supplementary Table S2). Of the 13, three were from pancreatic cancer patients, suggesting that PHASIFY MAX may improve mutation detection in low cfDNA-shedding tumors.

**Figure 4:**
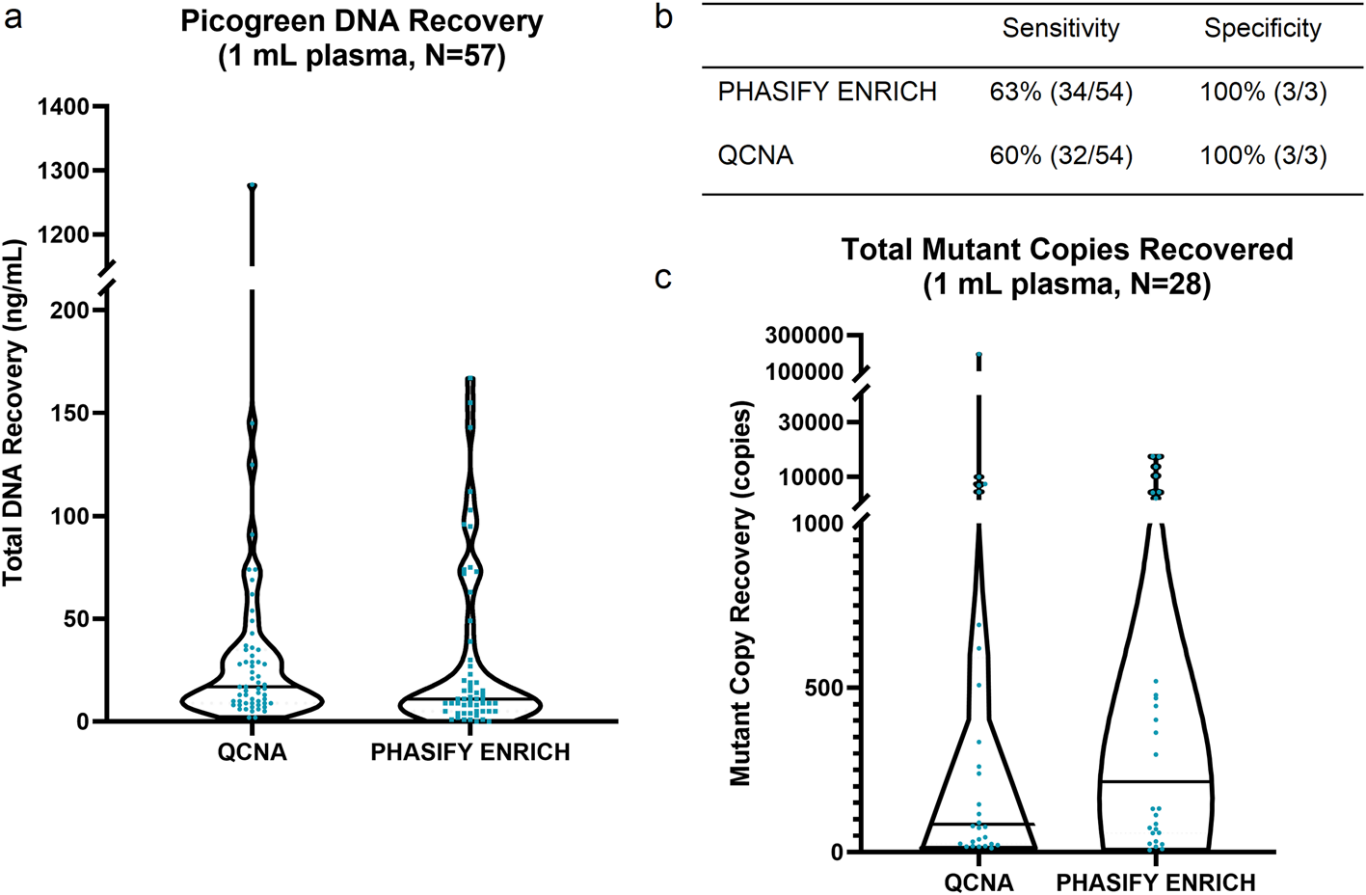
Results of PHASIFY ENRICH against QCNA. (**a**) A violin plot of total DNA recovery from 1 mL of plasma from QCNA and PHASIFY ENRICH. Median bars are shown as solid black lines. Median DNA recovery for QCNA and PHASIFY ENRICH are 17 ng and 11 ng, respectively (35% decrease). (**b**) Sensitivity and specificity values of PHASIFY ENRICH and QCNA against tissue genotyping. (**c**) A violin plot of total mutant copies recovered from QCNA and PHASIFY ENRICH out of the samples in which both kits detected mutations (n = 28). Median bars are shown as solid black lines. Median mutant copy recovery for QCNA and PHASIFY ENRICH are 85 copies and 215 copies, respectively (153% increase).

The PHASIFY ENRICH method resulted in median DNA isolation of 11 ng (range 0.2–167 ng), which is a 35% decrease compared to the median 17 ng (range 2.1–1,278 ng) DNA isolation of the reference QCNA method. However, the trend was not statistically significant (*P* = 0.28 Wilcoxon Signed-Rank) (Fig. 4a). This result is not surprising because PHASIFY ENRICH’s size selection feature is designed to remove genomic DNA, and the level of genomic DNA can vary greatly among patient samples. The ddPCR analysis of 57 samples extracted by PHASIFY ENRICH demonstrated clinical sensitivity and specificity (against tissue genotyping) of 63% (34/54) and 100% (3/3) respectively and 59% (32/54) and 100% (3/3) respectively with samples extracted by QCNA (Fig. 4b, Supplementary Table S2). Of the samples for which both methods confirmed mutations, on average, PHASIFY ENRICH achieved a 157% increase in recovery of mutant copies over the QCNA kit. In a median comparison of these data, PHASIFY ENRICH demonstrated a 153% increase compared to QCNA (*P* = 0.01 Wilcoxon Signed-Rank) (Fig. 4c).

### PHASIFY ENRICH follow-up clinical study

In a follow-up study, we tested a selected set of 47 plasma samples collected at different timepoints from 31 patients with diverse cancers that previously resulted in negative ddPCR cfDNA mutation status with QCNA extraction, despite known positive mutation status from corresponding tumor tissue genotyping (Supplementary Table S3). The elevated total DNA content in these samples when extracted with QCNA indicated the presence of high molecular weight contaminating DNA, which may have resulted in sub sampling concerns and ddPCR signal dilution. For this sample cohort, we focused only on the PHASIFY ENRICH method due to its ability to both recover greater cfDNA and reduce contaminating genomic DNA. With the combined advantages, we expected to observe mutant signal detection in this sample cohort. Using PHASIFY ENRICH, we isolated cfDNA from either 2 mL or 4 mL of plasma depending on remaining sample availability and compared ddPCR results to historical QCNA-extracted cfDNA isolated from 4 mL of plasma. The cfDNA mutation status was determined with probe-based (*BRAF*, *ESR1*, *IDH1*, *KRAS*, *NRAS*, *PIK3CA*, *TP53*) ddPCR. Specific probes were selected based on results of known tumor tissue genotyping. Of the 47 samples extracted by PHASIFY ENRICH, 9 samples resulted in a conversion to positive cfDNA mutation status compared to no mutant signals when using QCNA. Mutant allele fractions (MAFs) for the detected samples ranged between 0.1% and 36.2% (Supplementary Table S4). The PHASIFY ENRICH method recovered an average of 71.6 mutant copies / mL of plasma among the 9 detected samples.

## Discussion

Our results indicate that the PHASIFY MAX extraction technique offers higher cfDNA recovery resulting in better sensitivity of cfDNA mutation detection compared to solid-phase extraction represented by QCNA. Similarly, the PHASIFY ENRICH extraction technique demonstrated improved cfDNA mutation detection by increasing cfDNA recovery and reducing contaminating genomic DNA background. The improved signal-to-noise ratio resulted in the detection of mutant signals previously undetected in samples processed with QCNA. The mechanism of the improved recovery by liquid-phase extraction against solid-phase extraction has not been extensively studied. For solid-phase extraction methods, such as QCNA, theoretical cfDNA yield relies on the available surface area of the solid membrane and the subsequent elution efficiency. However, for liquid-phase extraction, cfDNA yield relies on the partitioning ability of the biomarker into the desired ATPS phase. We believe that the phase-forming ATPS domains result in greater capture of cfDNA compared to solid-phase methods. Further studies are required to confirm the potential benefit of liquid-phase partitioning over solid-phase extraction.

Liquid-phase extraction may also lead to advantages in automated sample preparation workflows. Automated workflows that utilize magnetic beads for cfDNA may capture encounter sample input inefficiencies due to the relatively large volume of magnetic beads required for extraction. The PHASIFY method uses smaller reagent volumes for sample processing, resulting in potential advantages in automation throughput and recovery. However, further studies are needed to evaluate the PHASIFY method for automated workflows.

There were certain limitations to this study. In particular, the sample cohorts were cancer agnostic and were not controlled for variables such as patient treatment status, cancer stage, or any other patient comorbidities that may affect cfDNA yield. Second, the initial study with clinical samples utilized only 1 mL of patient plasma, whereas many clinical diagnostic workflows extract 2–4 mL of plasma as standard practice. Due to the imposed limitation on sample input, the results are not representative of clinical sensitivities seen in real-world diagnostic applications. Further studies are required to evaluate PHASIFY performance with greater plasma input. Finally, in the PHASIFY ENRICH follow-up study, a period of 12 to 24 months had passed between the initial QCNA extraction and the PHASIFY ENRICH extraction, and all clinical samples underwent a freeze–thaw cycle in between the two extractions. As a result, potential cfDNA degradation may have occurred in the samples that were extracted with PHASIFY ENRICH.

The ability to (1) recover larger amounts of cf/ctDNA and (2) remove contaminating genomic DNA for downstream molecular diagnostics will be integral to increasing clinical sensitivity. In addition, by demonstrating mutation detection with half of the starting plasma volume as the QCNA solid-phase extraction, PHASIFY ENRICH’s procedure may avoid requiring large volume blood draws from patients. Overall, PHASIFY liquid extraction methods may contribute to unlocking additional cancer liquid biopsy applications including early stage therapy selection, recurrence monitoring and predictive screening.

## Methods

### Patients

Patients with advanced cancers receiving their care at MD Anderson Cancer Center participated in this study. The study was approved by the Institutional Review Board of MD Anderson (LAB10-0334). This study was conducted in accordance with the Declaration of Helsinki and written informed consent was obtained prior to enrollment.

### Blood processing and plasma collection

Approximately 20 mL of blood was collected from each patient in K2-ETDA tubes (Becton Dickinson (BD), Franklin Lakes, NJ). The blood samples were then centrifuged with a swing out rotor at 1,900 rcf (3,000 rpm) for 10 min at 4 °C. 8–9 mL of supernatant was then transferred to a clean 15 mL tube and centrifuged with a fixed-angle rotor at 16,000 rcf for 10 min at 4 °C. The plasma was then transferred to a clean Eppendorf cryogenic tube and stored at − 80 °C.

### PHASIFY MAX and ENRICH clinical specimens study design

91 plasma samples collected at different time points from 34 cancer patients with diverse advanced cancers were used in this retrospective study. Known *BRAF*, *KRAS*, or *NRAS* mutations were confirmed by tissue genotyping from a Clinical Laboratory Improvement Amendments (CLIA) certified laboratory. An initial cohort of N = 5 high-mutation rate samples was tested to confirm PHASIFY compatibility with the existing molecular diagnostic workflow. A second cohort of N = 47 samples was then tested to evaluate performance, followed by a third cohort of N = 39 samples to further evaluate performance with mostly low-mutation rate samples. Frozen plasma samples were thawed to room temperature, homogeneously mixed, and equally distributed into 1 mL aliquots. The cfDNA from each 1 mL aliquot was extracted by QIAamp Circulating Nucleic Acid extraction kit (QCNA; Qiagen, Hilden, Germany), PHASIFY MAX, or PHASIFY ENRICH according to the respective kit protocols. No modifications were made to the existing QCNA protocol. For valid comparison of the concentrations of extracted DNA, we ensured comparable elution volumes between the two kits (25 µL of elution buffer for PHASIFY kits and 30 µL of elution buffer for QCNA kits, accounting for an estimated 5 µL of lost volume in the spin column of QCNA).

### Overview of the PHASIFY MAX protocol

Briefly, 1 mL of plasma was mixed with protein digestion reagents supplied by the kit and incubated at 37 °C for 15 min. The plasma mixture was then added to the first provided ATPS and vortexed. The ATPS was centrifuged for 1 min at 7,000 rcf to engage phase separation. The bottom phase was then manually extracted via pipette and added to a second provided ATPS. The second ATPS was vortexed and centrifuged for 1 min at 7,000 rcf. Then 120 µL of the top phase was similarly extracted and added to an empty 2 mL microcentrifuge tube. The extract was mixed with the kit-provided salt solution, coprecipitant solution, and isopropanol and allowed to incubate for 5 min. The mixture was centrifuged for 10 min at 16,000 rcf. The supernatant was then removed, and the DNA pellet was washed three separate times with 1 mL of 40% isopropanol, 1 mL of 50% isopropanol, and 1 mL of cold 70% ethanol, in order. Each wash step was separated by a centrifugation step for 2 min at 16,000 rcf and removal of the supernatant. After removal of the 70% ethanol, the DNA pellet was dried at 56 °C for 15 min and resuspended in 25 µL of low ETDA TE buffer.

### Overview of the PHASIFY ENRICH protocol

Briefly, 1 mL of plasma was mixed with lysis reagents supplied by the kit and incubated at 37 °C for 15 min. The plasma mixture was then added to the first provided ATPS and vortexed. The ATPS was centrifuged for 1 min at 7,000 rcf to engage phase separation. The bottom phase was then manually extracted with a pipette and added to a second provided ATPS. The second ATPS was vortexed and centrifuged for 1 min at 7,000 rcf. The 120 µL of the top phase was similarly extracted and added to an empty 1.5 mL centrifuge tube. The extract was mixed with a proprietary fractionation solution and coprecipitant solution and incubated for 15 min. The solution was then centrifuged for 15 min at 16,000 rcf and the supernatant was added to an empty 2 mL microcentrifuge tube. The supernatant was mixed with the kit-provided salt solution, coprecipitant solution, and isopropanol and allowed to incubate for 5 min. The mixture was centrifuged for 10 min at 16,000 rcf. The supernatant was then removed, and the DNA pellet was washed three separate times with 1 mL of 40% isopropanol, 1 mL of 50% isopropanol, and 1 mL of cold 70% ethanol, in order. Each wash step was separated by a centrifugation step for 2 min at 16,000 rcf and supernatant removal. After removal of the 70% ethanol, the DNA pellet was dried at 56 °C for 15 min and resuspended in 25 µL of low ETDA TE buffer.

### Follow-up PHASIFY ENRICH clinical validation study design

47 plasma samples from 31 cancer patients with diverse advanced cancers were used for the follow-up experiment. Cancer patient disease status was confirmed with tissue positive genotyping (*BRAF*, *ESR1*, *IDH1*, *KRAS*, *NRAS*, *PIK3CA*, or *TP53*) by a CLIA certified laboratory. All plasma samples were previously determined negative for mutation status by ddPCR at MD Anderson Cancer Center. For the previous analysis, 4 mL of plasma sample was processed with QCNA as the DNA extraction method and the ddPCR input was capped at 16 ng. For the PHASIFY ENRICH extraction, either 2 mL or 4 mL of each sample (depending on remaining sample availability) was processed according to the kit protocol. Extracted DNA was eluted in 25 µL of PHASIFY elution buffer.

### Study analyses

Total DNA recovery was determined with the Quant-iT PicoGreen dsDNA Assay Kit (Invitrogen, Carlsbad, CA). 5 µL of each extracted sample was used for Picogreen analysis. For duplicate-well droplet digital PCR (ddPCR) analysis, 16 ng of extracted DNA was used as input whenever possible. If less than 16 ng of DNA was extracted from the plasma, then the entire DNA sample was added to the ddPCR reaction. 20 µL per well of reaction mix was added to the QX200 droplet generator (Bio-Rad). Each reaction mix contains 10 µL of 2 × probe super-mix, 1 µL of 20 × wildtype primer solution, 1 µL of 20 × mutant primer solution, and 8 µL of the cfDNA sample. The final concentrations of each primer (wildtype and mutant) are 900 nM. The final probe concentration is 250 nM. The samples were transferred to a 96 well-plate, sealed, and cycled in a C1000 Touch Thermal Cycler (Bio-Rad) using the following protocol: 95 °C for 10 min, followed by 39 cycles of 96 °C for 30 s and 55 °C for 1 min, followed by post-cycling steps of 98 °C for 10 min and a 12 °C hold. The cycled plate was then transferred to a QX200 ddPCR system reader.

2D ddPCR plots were generated for each sample (FAM plotted vs HEX) using QuantaSoft software (version 1.7.4; www.bio-rad.com). The new PHASIFY isolation methods resulted in clear separation between droplet clusters and were confirmed compatible with the ddPCR workflow (Supplementary Figure S1). The presence of three or more FAM-positive/HEX-negative (mutation-only) droplets in the 2D plot confirm positive mutation. The wildtype copies/µL and mutant copies/µL provided by the QuantaSoft software were used to determine MAF. The total mutant copies/mL of plasma was then calculated based on the mutant copies/µL and the amplifiable DNA concentration provided by ddPCR.

For the PHASIFY ENRICH follow-up clinical specimens study, 2D ddPCR plots were generated for each sample (FAM plotted vs HEX) using QuantaSoft software (version 1.7.4; www.bio-rad.com). The presence of three or more FAM-positive/HEX-negative (mutation-only) droplets with clear separation from the other droplet clusters in the 2D plot confirms positive mutation (Supplementary Figure S2). The PHASIFY ENRICH detection results were then compared to historical results from QCNA extraction. The wildtype copies/µL and mutant copies/µL provided by the QuantaSoft software were used to determine MAF. The mutant copies/mL of plasma was then calculated based on the mutant copies/µL and the amplifiable DNA concentration provided by ddPCR.

### Statistics and reproducibility

We performed a Wilcoxon signed-rank test to compare median differences in total DNA recovery and mutant copy recovery between QCNA and PHASIFY extraction on the same matched sample. Average yield improvement was calculated by determining the percent yield difference between PHASIFY and QCNA for each sample, and then averaging over all samples.

## Supplementary Information

Supplementary Information.

## References

[1] Polivka J, Pesta M, Janku F (2015). Testing for oncogenic molecular aberrations in cell-free DNA-based liquid biopsies in the clinic: Are we there yet?. Expert Rev. Mol. Diagn..

[2] Liu L (2018). Targeted methylation sequencing of plasma cell-free DNA for cancer detection and classification. Ann. Oncol..

[3] Janku F (2017). Development and validation of an ultradeep next-generation sequencing assay for testing of plasma cell-free DNA from patients with advanced cancer. Clin. Cancer Res..

[4] Bettegowda C (2014). Detection of circulating tumor DNA in early- and late-stage human malignancies. Sci. Transl. Med..

[5] Perkins G (2012). Multi-purpose utility of circulating plasma DNA testing in patients with advanced cancers. PLoS ONE.

[6] Janku F (2017). Multiplex KRAS G12/G13 mutation testing of unamplified cell-free DNA from the plasma of patients with advanced cancers using droplet digital polymerase chain reaction. Ann. Oncol..

[7] Lui YYN (2002). Predominant hematopoietic origin of cell-free DNA in plasma and serum after sex-mismatched bone marrow transplantation. Clin. Chem..

[8] Cohen JD (2018). Detection and localization of surgically resectable cancers with a multi-analyte blood test. Science.

[9] Diefenbach RJ, Lee JH, Kefford RF, Rizos H (2018). Evaluation of commercial kits for purification of circulating free DNA. Cancer Genet..

[10] Mashayekhi F, Meyer AS, Shiigi SA, Nguyen V, Kamei DT (2009). Concentration of mammalian genomic DNA using two-phase aqueous micellar systems. Biotechnol. Bioeng..

[11] Ribeiro SC, Monteiro GA, Cabral JMS, Prazeres DMF (2002). Isolation of plasmid DNA from cell lysates by aqueous two-phase systems. Biotechnol. Bioeng..

[12] Asenjo JA, Mistry SL, Andrews BA, Merchuk JC (2002). Phase separation rates of aqueous two-phase systems: Correlation with system properties. Biotechnol. Bioeng..

[13] Kamei DT (2002). Understanding viral partitioning in two-phase aqueous nonionic micellar systems: 1. Role of attractive interactions between viruses and micelles. Biotechnol. Bioeng..

[14] Pei Y, Wang J, Wu K, Xuan X, Lu X (2009). Ionic liquid-based aqueous two-phase extraction of selected proteins. Sep. Purif. Technol..

[15] Matos T, Johansson HO, Queiroz JA, Bulow L (2014). Isolation of PCR DNA fragments using aqueous two-phase systems. Sep. Purif. Technol..

[16] Newman AM (2014). An ultrasensitive method for quantitating circulating tumor DNA with broad patient coverage. Nat. Med..

[17] Chen Q, Zhang ZH, Wang S, Lang JH (2019). Circulating cell-free DNA or circulating tumor dna in the management of ovarian and endometrial cancer. Onco. Targets. Ther..

[18] Mody K, Cleary SP (2018). A review of circulating tumor DNA in hepatobiliary malignancies. Front. Oncol..

[19] Janku F (2016). BRAF Mutation testing in cell-free DNA from the plasma of patients with advanced cancers using a rapid, automated molecular diagnostics system. Mol. Cancer Ther..

[20] Riviere P (2018). The mutational landscape of gastrointestinal malignancies as reflected by circulating tumor DNA. Mol. Cancer Ther..

[21] Galot R (2020). Liquid biopsy for mutational profiling of locoregional recurrent and/or metastatic head and neck squamous cell carcinoma. Oral Oncol..

[22] Metcalf R (2017). The application of liquid biopsies in metastatic salivary gland cancer to identify candidate therapeutic targets. Ann. Oncol..

[23] Jensen K (2020). Detection of BRAFV600E in liquid biopsy from patients with papillary thyroid cancer is associated with tumor aggressiveness and response to therapy. J. Clin. Med..

[24] Sandulache VC (2017). Real-time genomic characterization utilizing circulating cell-free DNA in patients with anaplastic thyroid carcinoma. Thyroid.

