# Supplementary material for: A novel method for liquid-phase extraction of cell-free DNA for detection of circulating tumor DNA
Source: Sci Rep. 2021 Oct 4;11:19653. doi: 10.1038/s41598-021-98815-x (PMC8490367; doi:10.1038/s41598-021-98815-x)

A novel method for liquid-phase extraction of cell-free DNA for detection of circulating tumor DNA

Filip Janku[1*], Helen J. Huang[1], David Y. Pereira[2], Masae Kobayashi[2], Chung Hei Chiu[3], S. Greg Call[1], Kristen T. Woodbury[1], Felix Chao[2,3], Daniel R. Marshak[2,3], Ricky Y. T. Chiu[2,3]

[1] The University of Texas MD Anderson Cancer Center, Houston, TX, USA

[2] Phase Scientific International Ltd., Garden Grove, CA, USA

[3] Phase Scientific International Ltd., Kwun Tong, Hong Kong

SUPPLEMENTARY INFORMATION

Supplementary Table S1. PHASIFY MAX and PHASIFY ENRICH clinical specimens cohort.

	Sample (N, %)
Total Cohort	***91, 100%***
High cfDNA Shedding Cancers	***54, 59%***
Colorectal cancer	36, 40%
Tumor mutation status: BRAF V600E (13), KRAS G12/G13 (21), Wild-type (2)	
Non-small cell lung cancer	7, 8%
Tumor mutation status: BRAF V600E (7)	
Melanoma	4, 4%
Tumor mutation status: BRAF V600E (4)	
Cholangiocarcinoma	4, 4%
Tumor mutation status: BRAF V600E (4)	
Endometrial cancer	1, 1%
Tumor mutation status: Wild-type (1)	
Hepatocellular carcinoma	1, 1%
Tumor mutation status: Wild-type (1)	
Squamous cell cancer of head and neck	1, 1%
Tumor mutation status: Wild-type (1)	
Low cfDNA Shedding Cancers	***37, 41%***
Low grade serous ovarian cancer	18, 20%
Tumor mutation status: KRAS G12/G13 (10), NRAS Q61R (8)	
Appendiceal cancer	7, 8%
Tumor mutation status: BRAF V600E (5), KRAS G12/G13 (2)	
Pancreatic cancer	6, 7%
Tumor mutation status: KRAS G12/G13 (6)	
Papillary thyroid cancer	6, 7%
Tumor mutation status: BRAF V600E (6)	
PHASIFY MAX vs QCNA cohort	***89, 100%***
High cfDNA Shedding Cancers	***52, 58%***
Colorectal cancer	36, 40%
Tumor mutation status: BRAF V600E (13), KRAS G12/G13 (21), Wild-type (2)	
Non-small cell lung cancer	7, 8%
Tumor mutation status: BRAF V600E (7)	
Melanoma	4, 4%
Tumor mutation status: BRAF V600E (4)	
Cholangiocarcinoma	2, 2%
Tumor mutation status: BRAF V600E (2)	
Endometrial cancer	1, 1%
Tumor mutation status: Wild-type (1)	
Hepatocellular carcinoma	1, 1%
Tumor mutation status: Wild-type (1)	
Squamous cell cancer of head and neck	1, 1%
Tumor mutation status: Wild-type (1)	
Low cfDNA Shedding Cancers	***37, 42%***
Low grade serous ovarian cancer	18, 20%
Tumor mutation status: KRAS G12/G13 (10), NRAS Q61R (8)	

Appendiceal cancer	7, 8%
Tumor mutation status: BRAF V600E (5), KRAS G12/G13 (2)	
Pancreatic cancer	6, 7%
Tumor mutation status: KRAS G12/G13 (6)	
Papillary thyroid cancer	6, 7%
Tumor mutation status: BRAF V600E (6)	
PHASIFY ENRICH vs QCNA cohort	***57, 100%***
High cfDNA Shedding Cancers	***22, 39%***
Colorectal cancer	15, 26%
Tumor mutation status: KRAS G12/G13 (14), Wildtype (1)	
Cholangiocarcinoma	4, 7%
Tumor mutation status: BRAF V600E (4)	
Hepatocellular carcinoma	1, 2%
Tumor mutation status: Wild-type (1)	
Melanoma	1, 2%
Tumor mutation status: BRAF V600E (1)	
Squamous cell cancer of head and neck	1, 2%
Tumor mutation status: Wild-type (1)	
Low cfDNA Shedding Cancers	***35, 61%***
Low grade serous ovarian cancer	18, 32%
Tumor mutation status: KRAS G12/G13 (10), NRAS Q61R (8)	
Pancreatic cancer	6, 11%
Tumor mutation status: KRAS G12/G13 (6)	
Papillary thyroid cancer	6, 11%
Tumor mutation status: BRAF V600E (6)	
Appendiceal cancer	5, 9%
Tumor mutation status: BRAF V600E (3), KRAS G12/G13 (2)	

Supplementary Table S2. DNA isolation and mutation detection for each sample in the PHASIFY MAX and PHASIFY ENRICH clinical specimens study. Samples detected by PHASIFY MAX isolation but not detected by QCNA isolation (13 total) are denoted with an asterisk (*). Samples detected by PHASIFY ENRICH isolation but not detected by QCNA isolation (six total) are denoted with a dagger (†).

Tumor Type	Tumor Tissue Mutation	Sample ID	DNA Isolation by QCNA (from 1 mL plasma)	QCNA Mutation Detection (MAF)	DNA Isolation by PHASIFY MAX (from 1 mL plasma)	PHASIFY MAX Mutation Detection (MAF)	DNA Isolation by PHASIFY ENRICH (from 1 mL of plasma)	PHASIFY ENRICH Mutation Detection (MAF)
Appendiceal cancer	BRAF V600E	0101A	0.3	No Mutation	200.0	No Mutation	N/A	N/A
Appendiceal cancer	BRAF V600E	0101B†	29.0	No Mutation	728.0	No Mutation	27.0	0.21%
Appendiceal cancer	BRAF V600E	0101C	11.0	0.80%	49.0	0.64%	30.0	0.63%
Appendiceal cancer	BRAF V600E	0101D	2.2	2.90%	461.0	No Mutation	39.0	1.00%
Appendiceal cancer	BRAF V600E	0101E	1.3	4.10%	9.5	3.20%	N/A	N/A
Appendiceal cancer	KRAS G12D	0102A*†	22.0	No Mutation	16.0	0.35%	11.0	0.39%
Appendiceal cancer	KRAS G12C	0103A	35.0	No Mutation	18.0	No Mutation	11.0	No Mutation
Cholangiocarcinoma	BRAF V600E	0104A	5.8	6.60%	N/A	N/A	15.0	10.70%
Cholangiocarcinoma	BRAF V600E	0104B	7.9	3.20%	N/A	N/A	15.0	6.60%
Cholangiocarcinoma	BRAF V600E	0105A	17.0	2.90%	35.0	2.90%	23.0	31.30%
Cholangiocarcinoma	BRAF V600E	0105B	29.0	3.70%	135.0	1.33%	14.0	8.40%
Colorectal cancer	BRAF V600E	0106A	172.0	No Mutation	233.0	No Mutation	N/A	N/A
Colorectal cancer	BRAF V600E	0107A	1.7	No Mutation	90.0	No Mutation	N/A	N/A
Colorectal cancer	BRAF V600E	0107B	4.6	No Mutation	2.7	No Mutation	N/A	N/A
Colorectal cancer	BRAF V600E	0107C	0.3	No Mutation	2.4	No Mutation	N/A	N/A
Colorectal cancer	BRAF V600E	0107D	7.8	3.00%	1.8	No Mutation	N/A	N/A
Colorectal cancer	BRAF V600E	0107E	3.2	No Mutation	7.8	No Mutation	N/A	N/A
Colorectal cancer	BRAF V600E	0107F	1.2	No Mutation	1.7	No Mutation	N/A	N/A
Colorectal cancer	BRAF V600E	0107G*	3.1	No Mutation	1.2	2.10%	N/A	N/A

Colorectal cancer	BRAF V600E	0107H	3.6	No Mutation	12.2	No Mutation	N/A	N/A
Colorectal cancer	BRAF V600E	0107I	2.5	No Mutation	16.5	No Mutation	N/A	N/A
Colorectal cancer	KRAS G12D	0108A	10.0	15.40%	19.0	8.50%	N/A	N/A
Colorectal cancer	KRAS G12D/G13D	0109A	17.0	11.60%	12.0	12.40%	N/A	N/A
Colorectal cancer	KRAS G12V	0110A	18.0	0.36%	14.0	0.60%	3.0	No Mutation
Colorectal cancer	KRAS G12D	0111A	9.3	18.30%	11.0	18.60%	8.6	18.10%
Colorectal cancer	KRAS G12D	0111B	74.0	45.10%	12.0	45.40%	103.0	44.50%
Colorectal cancer	BRAF V600E	0112A	3.5	No Mutation	6.9	No Mutation	N/A	N/A
Colorectal cancer	BRAF V600E	0112B	1.6	No Mutation	4.3	No Mutation	N/A	N/A
Colorectal cancer	BRAF V600E	0112C	2.6	No Mutation	3.1	No Mutation	N/A	N/A
Colorectal cancer	KRAS G12C	0113A	54.0	43.00%	135.0	35.90%	73.0	47.70%
Colorectal cancer	KRAS G12C	0113B	1278.0	51.50%	538.0	48.50%	112.0	53.10%
Colorectal cancer	KRAS G12D	0114A	9.9	2.60%	61.0	0.96%	N/A	N/A
Colorectal cancer	KRAS G12D	0115A	74.0	19.60%	89.0	21.30%	75.0	18.70%
Colorectal cancer	KRAS G12D	0115B	69.0	36.90%	199.0	37.70%	155.0	36.10%
Colorectal cancer	KRAS G12D	0116A	272.0	No Mutation	862.0	No Mutation	N/A	N/A
Colorectal cancer	KRAS G12D	0116B*	2.9	No Mutation	14.0	0.25%	N/A	N/A
Colorectal cancer	KRAS G12D	0116C	7.0	No Mutation	16.0	No Mutation	N/A	N/A
Colorectal cancer	KRAS G12D	0116D	10.0	No Mutation	13.0	No Mutation	13.0	No Mutation
Colorectal cancer	KRAS G12D	0116E*	6.9	No Mutation	7.5	0.50%	3.9	No Mutation
Colorectal cancer	KRAS G12D	0116F*	15.0	No Mutation	21.0	0.35%	19.0	No Mutation
Colorectal cancer	KRAS G12D	0116G*	19.0	No Mutation	19.0	0.58%	0.2	No Mutation
Colorectal cancer	KRAS G12V	0117A	6.2	36.80%	24.0	15.60%	N/A	N/A
Colorectal cancer	KRAS G12V	0118A	145.0	No Mutation	824.0	No Mutation	74.0	No Mutation
Colorectal cancer	KRAS G12A	0119A*	10.0	No Mutation	16.0	0.24%	11.0	No Mutation
Colorectal cancer	WT	0120A	33.0	No Mutation	218.0	No Mutation	N/A	N/A
Colorectal cancer	WT	0121A	125.0	No Mutation	126.0	No Mutation	17.0	No Mutation
Colorectal cancer	KRAS G12D	0122A*	36.0	No Mutation	13.0	0.27%	9.0	No Mutation
Endometrial cancer	WT	0123A	10.0	No Mutation	48.0	No Mutation	N/A	N/A
Hepatocellular cancer	WT	0124A	91.0	No Mutation	143.0	No Mutation	143.0	No Mutation

Non-small cell lung cancer	BRAF V600E	0125A	2.8	No Mutation	11.0	No Mutation	N/A	N/A
Non-small cell lung cancer	BRAF V600E	0125B	3.5	No Mutation	6.4	No Mutation	N/A	N/A
Non-small cell lung cancer	BRAF V600E	0125C	8.2	No Mutation	7.1	No Mutation	N/A	N/A
Non-small cell lung cancer	BRAF V600E	0125D	7.2	No Mutation	4.2	No Mutation	N/A	N/A
Non-small cell lung cancer	BRAF V600E	0125E	35.0	No Mutation	347.0	No Mutation	N/A	N/A
Non-small cell lung cancer	BRAF V600E	0125F	6.5	No Mutation	17.0	No Mutation	N/A	N/A
Non-small cell lung cancer	BRAF V600E	0125G	6.3	No Mutation	8.6	No Mutation	N/A	N/A
Melanoma	BRAF V600E	0126A	8.7	23.50%	32.0	14.20%	8.5	5.30%
Melanoma	BRAF V600E	0126B	2.1	18.00%	4.3	13.70%	N/A	N/A
Melanoma	BRAF V600E	0126C	8.9	29.60%	10.6	24.90%	N/A	N/A
Melanoma	BRAF V600E	0127A	2.8	No Mutation	2.2	No Mutation	N/A	N/A
Low grade serous ovarian cancer	KRAS G12D	0128A*†	49.0	No Mutation	4.2	2.80%	0.9	5.30%
Low grade serous ovarian cancer	KRAS G12D	0128B	37.0	0.73%	221.0	No Mutation	7.8	3.60%
Low grade serous ovarian cancer	KRAS G12D	0128C	13.0	0.70%	12.0	0.70%	3.6	No Mutation
Low grade serous ovarian cancer	KRAS G12D	0128D	17.0	No Mutation	391.0	No Mutation	4.9	No Mutation
Low grade serous ovarian cancer	KRAS G12D	0128E*†	9.6	No Mutation	54.0	0.44%	4.6	1.40%
Low grade serous ovarian cancer	KRAS G12D	0128F	8.5	1.10%	2.4	No Mutation	0.6	No Mutation
Low grade serous ovarian cancer	KRAS G12D	0128G	21.0	0.21%	25.0	0.60%	12.0	0.27%
Low grade serous ovarian cancer	KRAS G12D	0128H	13.0	0.80%	21.0	0.97%	19.0	1.10%
Low grade serous ovarian cancer	KRAS G12D	0128I	5.5	2.30%	13.0	2.10%	15.0	1.30%
Low grade serous ovarian cancer	KRAS G12D	0128J	14.0	0.50%	14.0	1.10%	10.0	1.10%
Low grade serous ovarian cancer	NRAS Q61R	0129A	43.0	No Mutation	3.0	No Mutation	0.8	No Mutation

Low grade serous ovarian cancer	NRAS Q61R	0129B	35.0	No Mutation	7.3	No Mutation	1.6	No Mutation
Low grade serous ovarian cancer	NRAS Q61R	0129C	4.7	No Mutation	2.6	No Mutation	1.3	No Mutation
Low grade serous ovarian cancer	NRAS Q61R	0129D	7.0	1.20%	7.6	No Mutation	0.2	No Mutation
Low grade serous ovarian cancer	NRAS Q61R	0129E	11.0	0.72%	12.0	0.77%	4.9	0.50%
Low grade serous ovarian cancer	NRAS Q61R	0129F	5.6	0.70%	9.1	1.10%	5.4	1.60%
Low grade serous ovarian cancer	NRAS Q61R	0129G	9.2	0.70%	12.0	1.10%	5.3	1.10%
Low grade serous ovarian cancer	NRAS Q61R	0129H	13.0	1.90%	5.1	2.10%	3.6	2.30%
Pancreatic cancer	KRAS G12V	0130A*	8.4	No Mutation	33.0	0.35%	11.0	No Mutation
Pancreatic cancer	KRAS G12V	0130B	27.0	0.58%	123.0	0.84%	95.0	0.46%
Pancreatic cancer	KRAS G12V	0130C	16.0	4.90%	35.0	4.60%	49.0	3.20%
Pancreatic cancer	KRAS G12V	0131A*†	16.0	No Mutation	10.0	0.65%	8.8	0.37%
Pancreatic cancer	KRAS G12V	0131B	8.2	No Mutation	20.0	No Mutation	8.4	No Mutation
Pancreatic cancer	KRAS G12V	0131C*†	2.1	No Mutation	14.0	0.41%	8.7	1.00%
Squamous cell cancer of head and neck	WT	0132A	62.0	No Mutation	595.0	No Mutation	7.6	No Mutation
Thyroid cancer	BRAF V600E	0133A	32.0	No Mutation	55.0	No Mutation	72.0	No Mutation
Thyroid cancer	BRAF V600E	0133B	24.0	0.23%	88.0	0.29%	96.0	0.26%
Thyroid cancer	BRAF V600E	0133C	28.0	3.10%	67.0	3.40%	63.0	2.80%
Thyroid cancer	BRAF V600E	0133D	4.5	6.80%	35.0	6.20%	20.0	6.70%
Thyroid cancer	BRAF V600E	0133E	28.0	8.30%	236.0	7.50%	167.0	8.80%
Thyroid cancer	BRAF V600E	0134A	29.0	No Mutation	583.0	No Mutation	8.6	No Mutation

Supplementary Table S3. PHASIFY ENRICH follow-up study sample cohort with prior negative results for mutation detection in cell-free DNA.

	**Sample (N, %)**
Total Cohort	***47, 100%***
High cfDNA Shedding Cancers	***25, 53***%
Cholangiocarcinoma	8, 17%
Tumor mutation status: IDH1 R132C (4), IDH1 R132G (1), IDH1 R132S (1), KRAS G12/G13 (1), NRAS Q61 (1)	
Breast cancer	7, 15%
Tumor mutation status: ESR1 D538G (1), PIK3CA R545K (1), TP53 L265P (1), TP53 R273C (2), TP53 Y236C (2)	
Melanoma	5, 11%
Tumor mutation status: NRAS Q61 (4), KRAS (1)	
Non-small cell lung cancer	2, 4%
Tumor mutation status: KRAS G12C (2)	
Colorectal cancer	2, 4%
Tumor mutation status: PIK3CA H1047R (1), NRAS G12/G13 (1)	
Endometrial cancer	1, 2%
Tumor mutation status: PIK3CA H1047R (1)	
Low cfDNA Shedding Cancers	***22, 47%***
Adenoid cystic carcinoma of maxilla	7, 15%
Tumor mutation status: PIK3CA H1047R (5) IDH1 R132H (2)	
Pancreatic cancer	6, 13%
Tumor mutation status: KRAS G12/G13 (4), KRAS Q61 (1)	
Low grade serous ovarian cancer	4, 9%
Tumor mutation status: KRAS G12/G13 (3), BRAF V600E (1)	
Squamous cell cancer of head and neck	4, 9%
Tumor mutation status: TP53 R248Q (3) TP53 R273H (1)	
Papillary thyroid cancer	1, 2%
Tumor mutation status: BRAF V600E (1)	

Supplementary Table S4. Mutation detection in PHASIFY ENRICH follow-up study using samples with prior negative cfDNA mutation status when extracted with QCNA.

Cancer Type	Tumor Mutation	Sample ID	PHASIFY ENRICH mutation detection (MAF)
Breast cancer	TP53 L265P	0201A	0.26%
Breast cancer	PIK3CA R545K	0201B	0.70%
Breast cancer	TP53 Y236C	0202A	No Mutation
Breast cancer	TP53 Y236C	0202B	No Mutation
Breast cancer	ESR1 D538G	0202C	No Mutation
Breast cancer	TP53 R273C	0203A	1.60%
Breast cancer	TP53 R273C	0203B	No Mutation
Cholangiocarcinoma	KRAS G12/G13	0204A	No Mutation
Cholangiocarcinoma	IDH1 R132C	0205A	No Mutation
Cholangiocarcinoma	IDH1 R132C	0205B	0.75%
Cholangiocarcinoma	IDH1 R132S	0206A	0.42%
Cholangiocarcinoma	IDH1 R132C	0207A	No Mutation
Cholangiocarcinoma	IDH1 R132G	0208A	No Mutation
Cholangiocarcinoma	IDH1 R132C	0209A	No Mutation
Cholangiocarcinoma	NRAS Q61	0210A	No Mutation
Colorectal cancer	PIK3CA H1047R	0211A	No Mutation
Colorectal cancer	NRAS G12/G13	0212A	No Mutation
Adenoid cystic carcinoma of maxilla	IDH1 R132H	0213A	No Mutation
Adenoid cystic carcinoma of maxilla	IDH1 R132H	0213B	No Mutation
Adenoid cystic carcinoma of maxilla	PIK3CA H1047R	0214A	No Mutation
Adenoid cystic carcinoma of maxilla	PIK3CA H1047R	0214B	No Mutation
Adenoid cystic carcinoma of maxilla	PIK3CA H1047R	0214C	No Mutation

Adenoid cystic carcinoma of maxilla	PIK3CA H1047R	0214D	No Mutation
Adenoid cystic carcinoma of maxilla	PIK3CA H1047R	0214E	No Mutation
Endometrial cancer	TP53 R273H	0215A	No Mutation
Non-small cell lung cancer	KRAS G12C	0216A	No Mutation
Non-small cell lung cancer	KRAS G12C	0216B	No Mutation
Melanoma	KRAS Q61	0217A	36.20%
Melanoma	NRAS Q61	0218A	No Mutation
Melanoma	NRAS Q61	0218B	0.22%
Melanoma	NRAS Q61	0218C	No Mutation
Melanoma	NRAS Q61	0219A	No Mutation
Low grade serous ovarian cancer	BRAF V600E	0220A	No Mutation
Low grade serous ovarian cancer	KRAS G12/G13	0221A	No Mutation
Low grade serous ovarian cancer	KRAS G12V	0221B	No Mutation
Low grade serous ovarian cancer	KRAS G12V	0221C	No Mutation
Pancreatic cancer	KRAS G12/G13	0222A	0.10%
Pancreatic cancer	KRAS G12/G13	0223A	No Mutation
Pancreatic cancer	KRAS G12V	0224A	No Mutation
Pancreatic cancer	KRAS Q61	0225A	No Mutation
Pancreatic cancer	KRAS G12/G13	0226A	No Mutation
Pancreatic cancer	KRAS G12/G13	0227A	No Mutation
Squamous cell cancer of head and neck	TP53 R248Q	0228A	No Mutation
Squamous cell cancer of head and neck	TP53 R248Q	0228B	No Mutation
Squamous cell cancer of head and neck	TP53 R248Q	0229A	0.17%
Squamous cell cancer of head and neck	TP53 R273H	0230A	No Mutation
Thyroid cancer	BRAF V600E	0231A	No Mutation

Supplementary Table S5. Measurements of DNA purity from PHASIFY MAX and PHASIFY ENRICH kits. Plasma samples were spiked with a 145 bp dsDNA fragment and DNA was extracted using the PHASIFY MAX and PHASIFY ENRICH workflows. Final DNA was eluted in 25 µL of Low TE buffer. Absorbance measurements were conducted with the Nanodrop One. Triplicate extractions using each kit were conducted and triplicate UV absorbance measurements were conducted per extracted sample. Both extraction kits demonstrated adequate 260/280 ratios. The low 260/230 ratios are due to the proprietary DNA carrier reagents that aid DNA precipitation, which were found to absorb at λ=230 nm. The presence of the inert DNA carrier is found to not interfere with downstream qPCR or ddPCR diagnostics, as seen in Supplementary Figures S3 and S4.

Kit	Individual Sample 260/280 Ratio (n=3)	Average 260/280 Ratio	Individual Sample 260/230 Ratio (n=3)	Average 260/230 Ratio
**PHASIFY MAX**	1.78 ± 0.03	1.80 ± 0.07	0.03 ± 0.00	0.04 ± 0.01
	1.73 ± 0.03		0.04 ± 0.00	
	1.87 ± 0.04		0.03 ± 0.00	
**PHASIFY ENRICH**	1.61 ± 0.03	1.64 ± 0.03	0.17 ± 0.00	0.12 ± 0.06
	1.63 ± 0.02		0.13 ± 0.01	
	1.67 ± 0.03		0.05 ± 0.00	

Supplementary Figure S1. 2D ddPCR plots demonstrating mutation detection from QIAamp CNA, PHASIFY MAX, and PHASIFY ENRICH cfDNA isolation from three different patient samples in the clinical specimens cohort. Isolation by the PHASIFY method resulted in clear droplet separation between multiple channels, which indicates that the PHASIFY method is compatible with the ddPCR workflow. Blue droplets in FAM-positive/HEX-negative channels indicate detection of mutant copies and green droplets in FAM-negative/HEX-positive channels indicate detection of wild-type copies. The three samples were selected to represent typical droplet separation achieved by the PHASIFY method. The plots were generated using QuantaSoft version 1.7.4 (www.bio-rad.com).

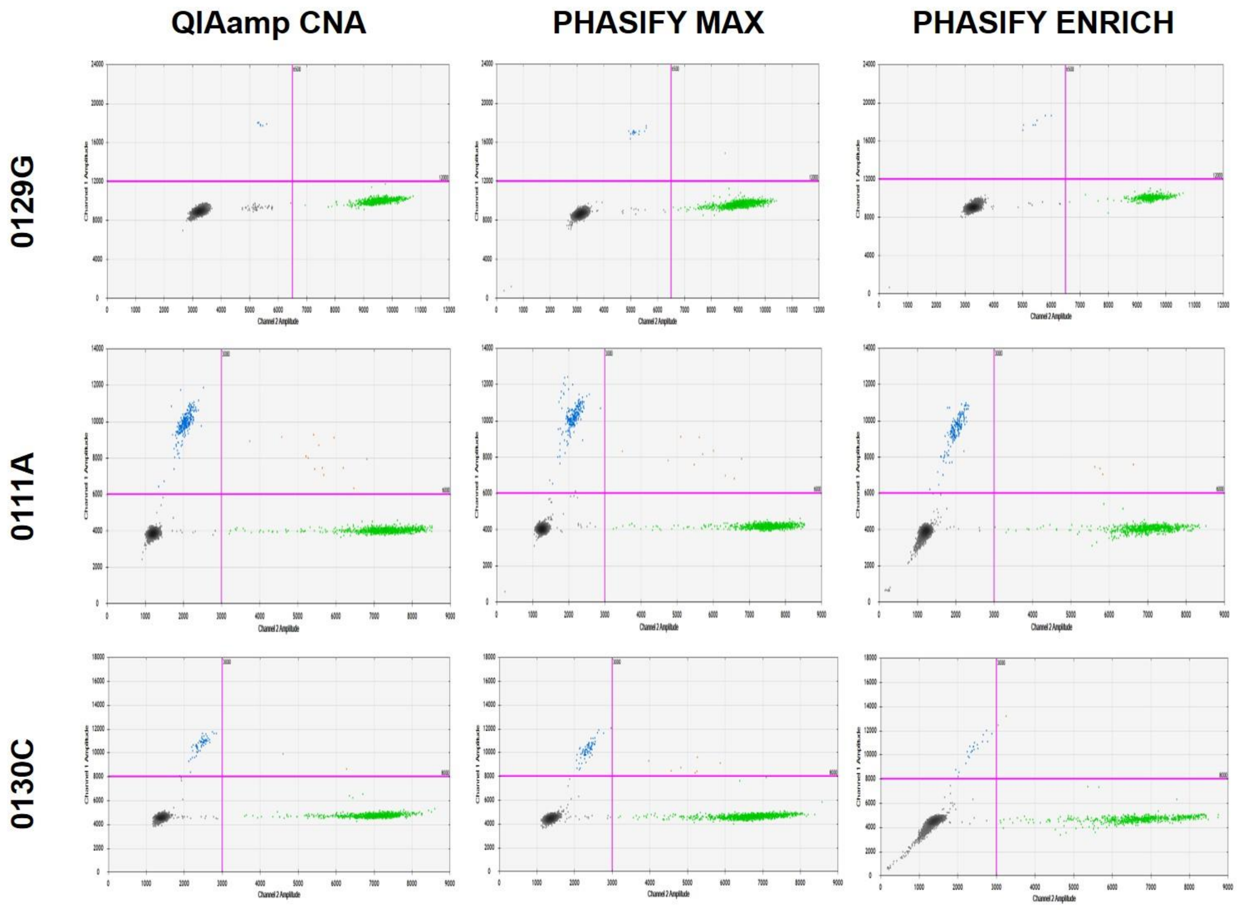

Supplementary Figure S2. 2D ddPCR plots demonstrating mutation detection from PHASIFY ENRICH cfDNA isolation from three different patient samples in a follow-up clinical specimens cohort. The same patient samples had prior negative cfDNA mutation status when cfDNA was isolated using QIAamp CNA (i.e., no blue FAM-positive/HEX-negative (mutation-only) droplets). The three samples were selected to represent typical droplet separation achieved by the PHASIFY method. The plots were generated using QuantaSoft version 1.7.4 (www.bio-rad.com).

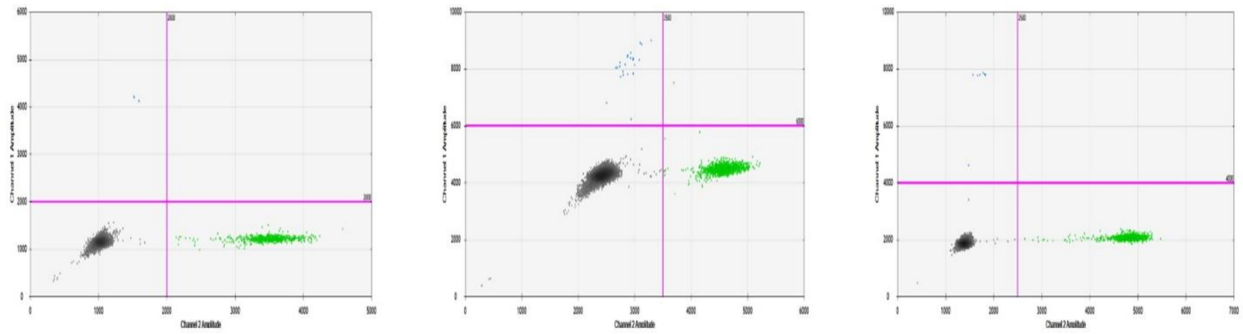

0206A **0203A** **0201B**

Supplementary Figure S3. Evaluation of potential qPCR inhibition of PHASIFY MAX and PHASIFY ENRICH. To investigate potential qPCR inhibition due to PHASIFY product eluents, varying volumes of fixed eluent were mixed with a fixed amount of 145 bp dsDNA fragments (2 µL of dsDNA at 50 pg/µL concentration) and applied to qPCR. The eluent from extractions with QCNA was used as a control. Eluent volumes ranged from 0 to 7 µL, with 7 µL being the maximum allowable input. The spiked cfDNA was detected using Taqman-based probe and primers specific for the 145 bp dsDNA sequence and quantified by qPCR using QuantStudio 3 (n=3). Cycle threshold (Cq) values remained consistent across the entire range of eluent inputs, which indicates that the PHASIFY kits do not inhibit qPCR.

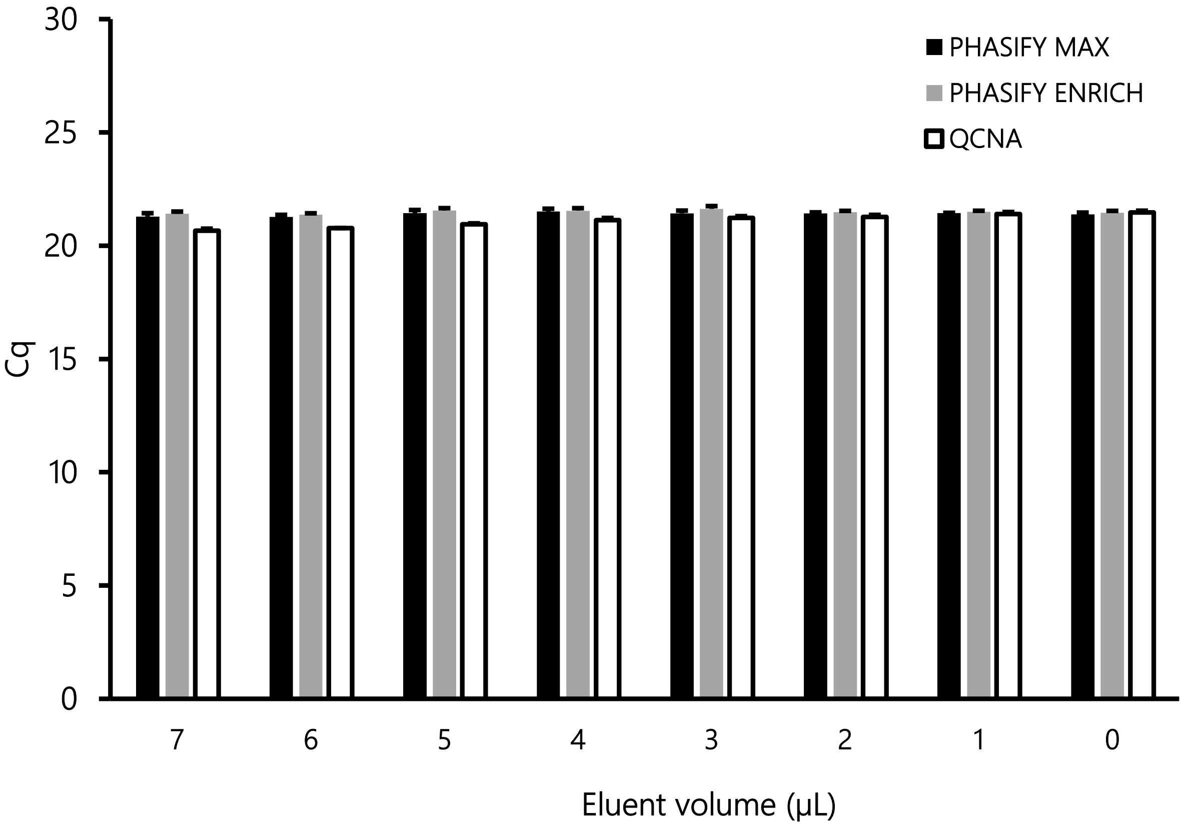

Supplementary Figure S4. Evaluation of potential ddPCR inhibition of PHASIFY MAX and PHASIFY ENRICH. To investigate potential ddPCR inhibition due to PHASIFY product eluents, varying volumes of fixed eluent were mixed with a fixed amount of 145 bp dsDNA fragments (2 µL of dsDNA at 0.2 fg/µL concentration) and applied to ddPCR. The eluent from extractions with QCNA was used as a control. Eluent volumes were 0, 3, 5, and 7 µL, with 7 µL being the maximum allowable input. The spiked cfDNA was detected using Taqman-based probe and primers specific for the 145 bp dsDNA sequence and quantified by ddPCR (Bio-Rad QX200). The recovered copy number remained consistent across the entire range of eluent inputs, which indicates that the PHASIFY kits do not inhibit ddPCR. Coefficient of variance (CV) among the different eluent volumes did not exceed 10% for any of the tested kits.

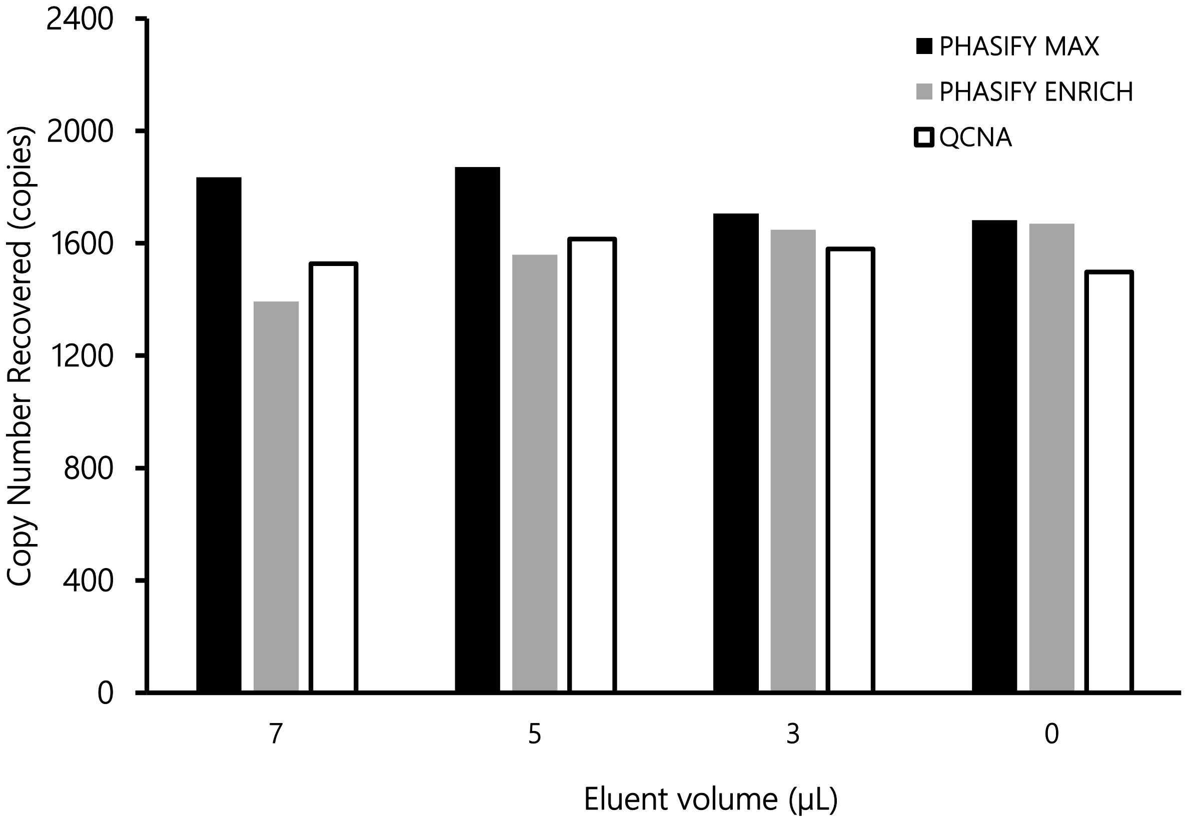

Supplement: Supplementary file 1 — Supplementary Information. [file 41598_2021_98815_MOESM1_ESM.pdf]
